# Superior mesenteric artery syndrome after colectomy: A case report and literature review

**DOI:** 10.1097/MD.0000000000030427

**Published:** 2022-09-02

**Authors:** Xiangmin Li, Min Tian, Hui Yang, Yulin Liu, Jingbo Chen, Hu Tian

**Affiliations:** a Master of Arts, Department of General Surgery, Shandong Provincial Qianfoshan Hospital, Shandong University, Jinan, Shandong, China; b Doctor of Medicine, Department of Nursing, Shandong Provincial Qianfoshan Hospital, Shandong University, Jinan, Shandong, China; c Doctor of Medicine, Department of General Surgery, Shandong Provincial Qianfoshan Hospital, Shandong University, Jinan, Shandong, China.

**Keywords:** Colectomy, Duodenal obstruction, Superior mesenteric artery syndrome

## Abstract

Superior mesenteric artery (SMA) syndrome, also known as Wilkie syndrome, is a rare benign disease characterized by small bowel obstruction due to compression of the third portion of the duodenum by the SMA and the abdominal aorta. However, SMA syndrome after colectomy is extremely rare, establishing a clear diagnosis and formulating a treatment plan may be challenging for surgeons.

**Patient concerns:**

A 64-year-old male with multiple colon polyposis and constipation underwent laparoscopic subtotal colectomy with cecal-rectal anastomosis. On the seventh postoperative day, he started vomiting and complained of abdominal bloating.

**Diagnosis:**

An upper gastrointestinal series, computed tomography scan and computed tomography angiography confirmed the diagnosis of SMA syndrome.

**Interventions:**

Gastric decompression, nasojejunal tube feeding and parenteral nutrition were performed.

**Outcomes:**

After 3 weeks of conservative treatment, the patient showed no clinical improvement in symptoms. Subsequently, he suffered from gastrointestinal hemorrhage, deep venous thrombosis of the lower extremity and cerebral hemorrhage successively. Unfortunately, the patient eventually died.

**Lessons:**

Surgeons should be aware of the fact that SMA syndrome can occur after colectomy. Every attempt should be made to correct and avoid any predisposing factors perioperatively. Prompt diagnosis of SMA syndrome after colectomy and appropriate early intervention reduce mortality.

## 1. Introduction

Superior mesenteric artery (SMA) syndrome, also known as Wilkie syndrome, is a rare benign disease characterized by small bowel obstruction due to compression of the third portion of the duodenum between the SMA and the abdominal aorta. Several catabolic pathological conditions that result in rapid weight loss (anorexia nervosa, sepsis, burns and multiple trauma) deplete the fat pad that separates the SMA from the aorta and may trigger the syndrome.^[1]^ It has also been reported in association with scoliosis surgery, abdominal aortic aneurysm repair and proctocolectomy with ileal pouch-anal anastomosis (IPAA).^[2]^

Colectomy is a common procedures for both benign and malignant diseases, such as multiple colon polyposis, constipation and cancer. However, SMA syndrome after colectomy is extremely rare. Here we report the first case of SMA syndrome after laparoscopic subtotal colectomy with cecal-rectal anastomosis and conduct a literature review.

## 2. Case report

A 64-year-old man presented to our hospital complaining of abdominal bloating and rare defecation (fewer than 2 bowel movements per week) for 5 years. He was 160 cm tall and weighed 44 kg (body mass index, 17.2 kg/m^2^). An endoscopic examination revealed multiple colon polyps (>100) mainly in the transverse, descending and sigmoid colon and several polyps larger than 2 cm in diameter. He was diagnosed as colonic slow transit constipation by colonic transit time test. There was no abnormal dilatation of the duodenum on preoperative computed tomography (CT) scan.

Laparoscopic subtotal colectomy was performed on the patient. After the entire colon was fully mobilized, the ileocecal valve and ileocolic vessels were preserved, and the majority of the right colon (the ascending colon 2–3 cm in length preserved distal to the ileocecal junction), transverse colon, left colon, and sigmoid colon were resected. The cecum was then rotated 180° around its original axis, with the ileocolic pedicle serving as the axis of rotation, and anastomosed to the rectum in an isoperistaltic manner.

**Figure 1.**
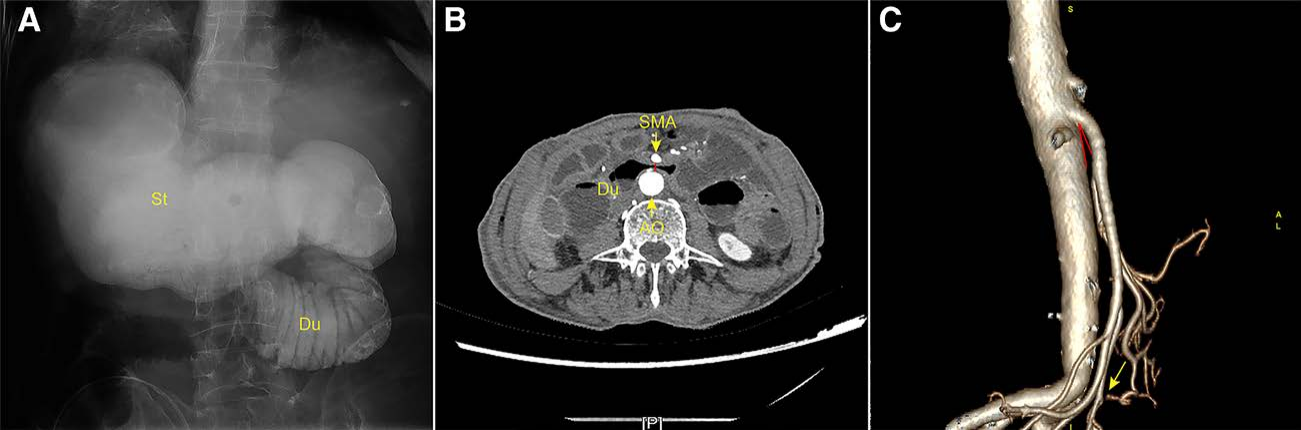
(A) Upper GI series showed a dilated stomach and proximal duodenum, a straight-line cutoff at the third part of the duodenum; (B) The distance between the aorta and SMA on CT was 4 mm; (C) The angle between the aorta and SMA on CT was 20°. Cecal-rectal anastomosis causes relative stretching of the SMA and flattens it against the aorta. Ao = aorta, Du = duodenum, SMA = superior mesenteric artery, St = stomach.

The patient experienced an uneventful immediate postoperative recovery and had oral nutrition reinstated on the fifth postoperative day. However, on the seventh postoperative day, he complained of abdominal discomfort and bloating, sudden onset of vomiting ensued. Decompression of the gastrointestinal (GI) tract was performed, large amounts of bilious fluid was drained through nasogastric tube. An upper GI (UGI) series showed a dilated stomach and proximal duodenum, a straight-line cutoff at the third part of the duodenum, to-and-fro peristalsis of the contrast, and the contrast went slowly through the third part of the duodenum (Figure 1A). A CT scan of the abdomen revealed distention of the stomach and the duodenum, abnormal narrowing of the aortomesenteric distance to 4 mm (Figure 1B). A CT angiography demonstrated an abnormally decreased aortomesenteric angle measuring 20° (Figure 1C). Imaging studies confirmed the diagnosis of SMA syndrome. A nasojejunal feeding tube guided by X-ray was advanced beyond the Treitz ligament to provide enteral alimentation. Nasogastric decompression, parenteral nutrition was continued and various positions encouraged. The patient cannot tolerate large amounts of enteral nutrition. After 3 weeks of conservative treatment, the patient showed no clinical improvement in symptoms. In view of postoperative intra-abdominal inflammation, adhesion and poor condition, a further laparotomy was not performed. Subsequently, the patient suffered from GI hemorrhage, endoscopic examination showed a 15-mm diameter, reddish soft round protruding submucosal mass with bleeding on the surface in the lower esophagus. There was no evidence of hemorrhage in the stomach and duodenum. Hemangioma of the esophagus was confirmed. The bleeding was controlled with endoscopically placed clips that were deployed at the site of the bleed. Next, he complained of leg swelling and pain, deep venous thrombosis was detected by ultrasonography and treated by subcutaneous low-molecular-weight heparin. Unfortunately, the patient suffered a sudden loss of consciousness and eventually died of cerebral hemorrhage.

## 3. Discussion and Conclusions

SMA syndrome, also known as Wilkie syndrome or benign duodenal stasis, is a rare but severe condition associated with the compression of the third portion of the duodenum between the SMA and the abdominal aorta causing abdominal pain and vomiting.

Severe catabolic conditions that cause marked weight loss (anorexia nervosa, sepsis, burns and multiple trauma) deplete the fat pad that separates the SMA from the aorta and may trigger the syndrome. It can also be associated with conditions known to cause reduced aortomesenteric angle like scoliosis surgery, abdominal aortic aneurysm repair and proctocolectomy with IPAA.^[2]^ The underlying predisposing anatomical risk factors include a low origin of the SMA artery, high insertion of the ligament of Treitz and acute aortomesenteric angle.^[3]^ To date, only 2 cases after colectomy were reported in English literatures.^[4,5]^ The reports are shown in Table 1. According to the reported cases, including the present case, left hemicolectomy, right hemicolectomy and subtotal colectomy was performed respectively, the median age of patients with SMA syndrome was 70 (range 64–79) years. Two patients presented with weight loss or malnutrition, but the first obese patient was observed after colectomy.^[4]^ A similar etiology is seen in patients who develop SMA syndrome following colectomy, the condition is associated with increasing the tension on the mesentery and narrowing aortomesenteric angle caused by stretching the SMA, ileocolic artery or middle colic artery. Middle colic artery may be involved in vascular compression of the duodenum due to hyper-mobility of the right colon without the fixation of hepatic flexure. Secondly, loss of fat pad in the retroperitoneum narrows the aortomesenteric angle. In the normal individual, the fat pad displaces the SMA anteriorly away from the aorta so avoiding duodenal compression by increasing the space for the duodenum to pass. Loss of this retroperitoneal fat narrows the aortomesenteric angle resulting in functional obstruction. Chronic wasting conditions such as malabsorption, cancer, ulcerative colitis, and eating disorders caused by intractable constipation can lead to weight loss. Similarly, rapid weight loss can be detected after surgery, such as bariatric surgery, esophagectomy, and other abdominal surgery. Once SMA syndrome occurs, food intolerance with nausea and vomiting exacerbates further weight loss, which may further reduce retroperitoneal fat and aggravate the condition, resulting in a vicious cycle.

**Table 1:** Superior mesenteric artery syndrome after colectomy: cases in the literature.

	Disease	Surgery	Diagnostic method	Treatment		Outcome
				Conservative	Surgical	
Yerdel et al.^[4]^ 1992	Synchronous descending and sigmoid colon cancer	Left hemicoletomy	UGI series	Nasogastric tube, parenteral nutrition	No	Good
Fearon et al.^[5]^ 2013	Ascending colon cancer	Right hemicoletomy	Contrast CT	Nasogastric tube, parenteral nutrition, nasojejunal tube	No	Good
Our study. 2022	Multiple colon polyposis and constipation	Subtotal colectomy with cecal-rectal anastomosis	UGI series, contrast CT	Nasogastric tube, parenteral nutrition, nasojejunal tube	No	Dead

CT = computed tomography; UGI = upper gastrointestinal.

The diagnosis of SMA syndrome remains challenging as other diseases present similar symptoms including intermittent postprandial abdominal pain, bloating, nausea, and bilious vomiting. The diagnosis is mainly based on high index of clinical suspicion and confirmed by imaging studies. UGI series may demonstrate dilatation of the proximal duodenum with failure of contrast passage through the third part of the duodenum with a cutoff.^[6]^ CT angiography can not only show the narrowed aortomesenteric angle of <25° and diminution of the aortomesenteric distance to <10 mm,^[7]^ but also the extent of duodenal obstruction.

Conservative management is the first line of therapy for SMA syndrome and was shown to be effective in 2 cases described,^[4,5]^ which includes gastric decompression, correction of electrolyte abnormalities, nasojejunal tube feeding, and encourage small regular feedings in the form of a clear liquid diet once the patient can tolerate oral intake and positional changes, such as the knee-chest or left lateral decubitus position after eating. Parenteral nutrition with high-calorie supplements is also an option.

Surgery is indicated if conservative management fails. Surgical procedures include duodenojejunostomy, gastrojejunostomy, and Strong procedure involving division of the ligament of Treitz and displacement of the duodenum downward and away from the apex of the aortomesenteric angle.^[1]^ Laparoscopic duodenojejunostomy to bypass the third part of the duodenum has recently become a reliable and useful method,^[8]^ particularly due to the improvements in minimally invasive surgery. Based on the experience of our case, if the patient cannot tolerate enteral nutrition, nutrient intake is inadequate, the symptoms is severe and the prognosis is unclear, prompt reoperation is considered. Bleeding from hemangioma of the esophagus might be associated with prolonged friction between the nasogastric tube and the hemangioma. Ineffective nutritional management and other complications might affect the treatment of SMA syndrome. Reoperation in the early postoperative setting may be the key to improve the cure rate and reduce mortality. Gastrojejunostomy may be ideal when the patients present with severe duodenal or gastric dilatation, or when it is difficult to mobilize the duodenum like our case.

There is obviously difficulty in foreseeing which patients are in danger of developing SMA syndrome after surgery so that further endeavors might be necessary for prevention. In our practice, in addition to tension-free anastomosis, routine Strong procedure should be proposed in emaciated patients of left hemicolectomy or total colectomy since it helps avoid enterotomy. Intestinal derotation is suggested by some surgeons for extended colon resection to avoid excessive traction.^[9]^ For patients with UGI obstruction (vomiting and difficulty eating), UGI imaging should be performed before surgery to exclude SMA syndrome. Nasojejunal tube facilitates enteric feeding and weight gain in select patients with marked weight loss preoperatively that might prevent the SMA syndrome. SMA syndrome is associated with malnutrition that is exacerbated by additional weight loss during hospitalization and is due in part to the substantial catabolic response to surgery. Appropriate preoperative nutrition can reduce complications, shorten hospitalization and improve postoperative outcomes. International guidelines recommend nutritional support for severely malnourished patients 7 to 14 days before elective major surgery.^[10]^ Delaying surgery to institute nutritional support preoperatively is necessary for patients with body mass index <18.5 kg/m^2^ or serious hypoproteinemia. Enteral nutrition is preferred for malnourished and aged patients.

In conclusion, surgeons should be aware of the fact that SMA syndrome can occur after colectomy in a similar manner to that reported after proctocolectomy and IPAA. Every attempt should be made to correct and avoid any predisposing factors perioperatively. Prompt diagnosis of SMA syndrome after colectomy and appropriate early intervention reduce mortality.

## Author contributions

Conceptualization: Min Tian and Hu Tian; writing-original draft preparation: Xiangmin Li; data collection and analysis: Hui Yang, Yulin Liu, Jingbo Chen; writing-review and editing: Min Tian; funding acquisition: Min Tian and Hu Tian. All authors read and approved the final manuscript.
